# Treatment of COVID-19 pneumonia with glucocorticoids (CORTIVID): a structured summary of a study protocol for a randomised controlled trial

**DOI:** 10.1186/s13063-020-04999-4

**Published:** 2021-01-11

**Authors:** Iñigo Les Bujanda, Jose Loureiro-Amigo, Ferran Capdevila Bastons, Iñaki Elejalde Guerra, Javier Anniccherico Sánchez, Anna Murgadella-Sancho, Ruth García Rey, Julián Librero López, Julio Sánchez Álvarez

**Affiliations:** 1 Internal Medicine Department, Complejo Hospitalario de Navarra, Pamplona, Spain; 2 Internal Medicine Department, Hospital Moisès Broggi, Consorci Sanitari Integral, Sant Joan Despí, Barcelona, Spain; 3 Hospital Pharmacy Department, Complejo Hospitalario de Navarra, Pamplona, Spain; 4 Hospital Pharmacy Department, Hospital Moisès Broggi, Consorci Sanitari Integral, Sant Joan Despí, Barcelona, Spain; 5 Clinical Trials Platform, Navarrabiomed, Complejo Hospitalario de Navarra, Universidad Pública de Navarra, Instituto de Investigación Sanitaria de Navarra, Pamplona, Spain; 6 Methodology Unit, Navarrabiomed, Complejo Hospitalario de Navarra, Universidad Pública de Navarra, Instituto de Investigación Sanitaria de Navarra, Pamplona, Spain

**Keywords:** COVID-19, Randomised controlled trial, protocol, SARS-CoV-2, Pneumonia, Quadruple blind, Glucocorticoids, Methylprednisolone, Pulses, Early treatment

## Abstract

**Objectives:**

The aim of this study is to assess the effectiveness and safety of glucocorticoid infusion pulse therapy to improve the clinical outcomes of patients with COVID-19 pneumonia with elevated inflammatory biomarkers.

**Trial design:**

A parallel-group quadruple-blind (participant, intervention provider, outcome assessor and data manager), randomised controlled trial.

**Participants:**

All patients admitted to hospital due to COVID-19 pneumonia will be considered eligible. Potential candidates will be identified and consecutively included in the emergency room or in the COVID-19 admission wards of two hospitals in Spain: Complejo Hospitalario de Navarra (Pamplona) and Hospital Moisès Broggi (Sant Joan Despí, Barcelona).

Inclusion criteria are: 1) age ≥18 years old; 2) diagnosis of SARS-CoV-2 pneumonia confirmed by reverse transcriptase polymerase chain reaction (RT-PCR) of nasopharyngeal swabs or sputum in accordance with the recommendations of the Spanish Ministry of Health; 3) history of symptoms compatible with COVID-19 ≥7 days; 4) hospital admission; 5) at least one of the following: C-reactive protein (CRP) >60 mg/dL, interleukin-6 (IL-6) >40 pg/mL, and/or ferritin >1000 μg/L; and 6) provision of informed consent.

Exclusion criteria are: 1) allergy or contraindication to any of the drugs under study; 2) oxygen saturation (SpO_2_) <90% (in air ambient) or partial pressure of oxygen in arterial blood (PaO_2_) <60 mmHg (in ambient air) or PaO_2_/FiO_2_ <300 mmHg; 3) ongoing treatment with glucocorticoids, or other immunosuppressants, including biologics for another indication; 4) decompensated diabetes mellitus; 5) uncontrolled hypertension; 6) psychotic or manic disorder; 7) active cancer; 8) pregnancy or breastfeeding; 9) clinical or biochemical suspicion (procalcitonin >0.5 ng/mL) of active infection other than with SARS-CoV-2; 10) management as an outpatient; 11) conservative or palliative management; 12) participation in another clinical trial; or 13) any major uncontrolled medical, psychological, psychiatric, geographic or social problem that contraindicates the patient's participation in the trial or hinders proper follow-up and adherence to the protocol and evaluation of study outcomes.

**Intervention and comparator:**

Eligible patients will be randomised to receive standard of care plus methylprednisolone (intervention group) or standard of care plus placebo (control group).

- Intervention group: standard of care at the discretion of the researcher, including lopinavir/ritonavir (200/50 mg, 2 tablets twice daily, per os, for 7 to 14 days) ± remdesivir (a single intravenous loading dose of 200 mg on day 1 followed by once-daily intravenous maintenance doses of 100 mg from day 2 to 5), or no drug treatment, + methylprednisolone (once-daily intravenous infusion of 120 mg on days 1, 2 and 3).
- Control group: standard of care at the discretion of the researcher, including lopinavir/ritonavir (200/50 mg, 2 tablets every 12 hours, per os, for 7 to 14 days) ± remdesivir (a single intravenous loading dose of 200 mg on day 1 followed by once-daily intravenous maintenance doses of 100 mg from day 2 to 5), or no drug treatment, + placebo (once-daily intravenous infusion of 100 mL of 0.9% saline on days 1, 2 and 3).

**Main outcomes:**

The primary outcome is the proportion of patients with treatment failure at 14 days after randomisation, defined as: 1) death, 2) need for admission to an intensive care unit (ICU), 3) initiation of mechanical ventilation, 4) SpO_2_ falling to <90% (in ambient air) or PaO_2_ <60 mmHg (in ambient air) or PaO_2_FiO_2_ <300 mmHg, not explained by a cause other than COVID-19, and/or 5) decrease in PaO_2_ ≥15% from baseline, together with laboratory and radiological deterioration.

**Randomisation:**

Treatment will be allocated by block randomisation stratified by patient age (< or ≥ 75 years of age). For this purpose, we will use the R randomizeR package using two block sizes (4 and 6) with random permutation. The randomisation sequence will be generated by a unit (the Navarrabiomed Clinical Trials Platform) independent from the researchers who will recruit patients and implement the protocol.

**Blinding (masking):**

The study will be quadruple-blinded, specifically, with blinding of patients, intervention providers, outcome assessors and data managers. The pharmacy at each participating hospital will prepare indistinguishable bags of methylprednisolone or placebo (0.9% saline) for patients of the experimental and placebo groups, respectively.

**Numbers to be randomised (sample size):**

The percentage of patients with treatment failure (primary endpoint) is currently unknown. Assuming an absolute difference of 25% in the primary outcome between the two groups (35% in the control group and 10% in the intervention group), we estimate that 60 patients (30 per group) are required to detect this difference with a two-tailed type I error of 0.05 and a type II error of 0.2. Estimating a loss to follow-up of 20%, we should recruit a total sample size of 72 patients (36 per group).

**Trial Status:**

The Spanish Agency of Medicines and Medical Devices (AEMPS) and the Ethics Committee of the University Hospital La Princesa approved version 7.0 of the protocol on 30 April 2020 as a low intervention clinical trial. Subsequently, the protocol has been amended by researchers and re-approved by AEMPS and the same ethics committee on 1 July 2020 (version 8.0) and on 28 August 2020 (version 9.0). Currently, the trial is in the recruitment phase. Recruitment began on 28 May 2020 and is expected to be completed by February 2021.

**Trial registration:**

This study protocol was registered on the eudract.ema.europa.eu on 5 May 2020 (title "Early treatment of COVID-19 pneumonia with glucocorticoids. Randomized controlled clinical trial"; EudraCT Number: 2020-001827-15) and on clinicaltrials.gov on 19 June 2020 (title: "Glucocorticoids in COVID-19 (CORTIVID)"; identifier: NCT04438980).

**Full protocol:**

The full protocol (version 9.0) is attached as an additional file, accessible from the Trials website (Additional file 1). In the interest in expediting dissemination of this material, the familiar formatting has been eliminated; this Letter serves as a summary of the key elements of the full protocol.

**Supplementary Information:**

The online version contains supplementary material available at 10.1186/s13063-020-04999-4.

## Supplementary Information

**Additional file 1.** Full Protocol.

## Data Availability

Not applicable.

