# Supplementary material for: Treatment of COVID-19 pneumonia with glucocorticoids (CORTIVID): a structured summary of a study protocol for a randomised controlled trial
Source: Trials. 2021 Jan 11;22:43. doi: 10.1186/s13063-020-04999-4 (PMC7797701; doi:10.1186/s13063-020-04999-4)
Supplement: Supplementary file 1 — Additional file 1. Full Protocol. [file 13063_2020_4999_MOESM1_ESM.docx]

**TREATMENT OF COVID-19 PNEUMONIA WITH GLUCOCORTICOIDS. A RANDOMISED CONTROLLED TRIAL**

(Version 9.0; 20th July 2020)

Protocol code number: CORTIVID

EudraCT number: 2020-001827-15

Principal investigator and coordinator:

Dr. Iñigo Les Bujanda. Complejo Hospitalario de Navarra. C/ Irunlarrea 3, 31008 – Pamplona, Spain.

Collaborating investigators:

Dr. Jose Loureiro-Amigo, Anna Murgadella-Sancho. Hospital Moisès Broggi, Consorci Sanitari Integral. C/Oriol Martorell 12, 08970 – Sant Joan Despí, Barcelona, Spain.

Ferran Capdevila Bastons, Dr. Iñaki Elejalde Guerra, Dr. Javier Annicchericho Sánchez, Dr. Julio Sánchez Álvarez. Complejo Hospitalario de Navarra. C/ Irunlarrea 3, 31008 – Pamplona, Spain.

Lead trial methodologist:

Julián Librero López. Navarrabiomed. C/ Irunlarrea 3, 31008 – Pamplona, Spain.

Clinical trial coordinator and pharmacovigilance:

Ruth García Rey. Navarrabiomed. C/ Irunlarrea 3, 31008 – Pamplona, Spain.

Name of sponsor: Navarrabiomed-Fundación Miguel Servet

C/ Irunlarrea 3, 31008 – Pamplona, Spain

**1.- BACKGROUND AND RATIONALE**

**1.1 BACKGROUND**

Coronaviruses are an important group of pathogenic viruses for humans and other animals. At the end of 2019, a new type of coronavirus was identified, SARS-CoV-2, following an outbreak of pneumonia in Wuhan City, Hubei Province, China. The disease caused by SARS-CoV-2 has been called Coronavirus Disease 2019 (COVID-19), and the outbreak turned rapidly into an epidemic that spread through China and across the rest of the world, with cases having been described in all the continents except Antarctica (1). To date, there is no effective evidence-based treatment for COVID-19. Hence, treatment recommendations are based on clinical experience and pilot studies with small numbers of patients, considerable methodological weaknesses and several schools of thought.

The focus in the search for new treatments for COVID-19 has been on antiviral drugs and vaccines. Current data suggest that the disease has three stages (Figure 1): an early infection phase related to the viral response (stage I), lasting 5 to 7 days; a pulmonary phase (stage II), up to 10 to 12 days after the onset of symptoms; and finally, in a subgroup of patients, a hyperinflammation phase (stage III), consisting of cytokine release syndrome that tends to lead to acute respiratory distress syndrome (ARDS), invasive mechanical ventilation almost always being required to keep the patient alive, as well as to other complications such as hemophagocytic and antiphospholipid antibody syndromes (2, 3).

The main cause of death in COVID-19 is acute respiratory failure secondary to ARDS, triggered by the viral infection in the hyperinflammation phase (4). It is known that both the innate and adaptive immune systems are involved in this response, leading to T-cell activation and differentiation and the release of type 1 interferons and pro-inflammatory cytokines (IL-1, IL-6, IL-8, IL-21, TNF-β and MCP-1), among other immune mediators (5).

During the previous pandemics caused by coronaviruses (severe acute respiratory syndrome [SARS] and the Middle East respiratory syndrome [MERS]), glucocorticoids were not among the recommended evidence-based treatments, as it seemed that they could be associated with a poorer course of the disease (6). In line with this, the World Health Organisation (WHO) advised against the use of glucocorticoids in the treatment of SARS-CoV-2, given that some studies found these drugs to be associated with a delayed viral clearance, psychosis, complications related to diabetes mellitus, avascular necrosis and even a higher risk of mortality (7).

Despite this recommendation by the WHO, given the poor response to antiviral drugs and growing data on the cytokine release syndrome in COVID-19, the medical community has started to prescribe glucocorticoids empirically in the early inflammatory stage of the disease. Their use in real clinical practice has been supported by more recent publications suggesting that glucocorticoids do benefit patients without significant adverse effects or delayed viral clearance (8, 9), and in Spain, by the inclusion of these drugs in the majority of hospital protocols for COVID-19. Nonetheless, the level of evidence available leaves many questions unanswered, that are difficult to address outside of a comparative clinical trial.

From the molecular point of view, pulsed glucocorticoids over 3 to 5 days at high doses (from 100 mg/day of prednisone or equivalent) work through dual (genomic and non-genomic) mechanisms of action with a low risk of toxicity. That is, as well as strong genomic effects following the complete saturation of the cytosolic glucocorticoid receptors (GRs) and their subsequent translocation into the cell nucleus, high doses lead to non-genomic effects mediated by selective membrane receptors (10). These additional non-genomic effects are associated with strong clinical response that is rapid, as it does not depend on the process of nuclear translocation of the glucocorticoid-GR complex and modulation of gene expression. On the other hand, the majority of adverse events related to glucocorticoids, including infections, are dose- and time-dependent, and hence tend to be associated with long-term maintenance treatments at doses under 100 mg/day of prednisone or equivalent that only activate the genomic pathway.

Given all this, the objective of this study was to assess the safety and efficacy of pulsed glucocorticoids in the inflammation phase (stage II) of COVID-19 for modifying the clinical course of the disease, need for mechanical ventilation, intensive care unit (ICU) admission and mortality.

**Figure 1. Phases of COVID-19**

**
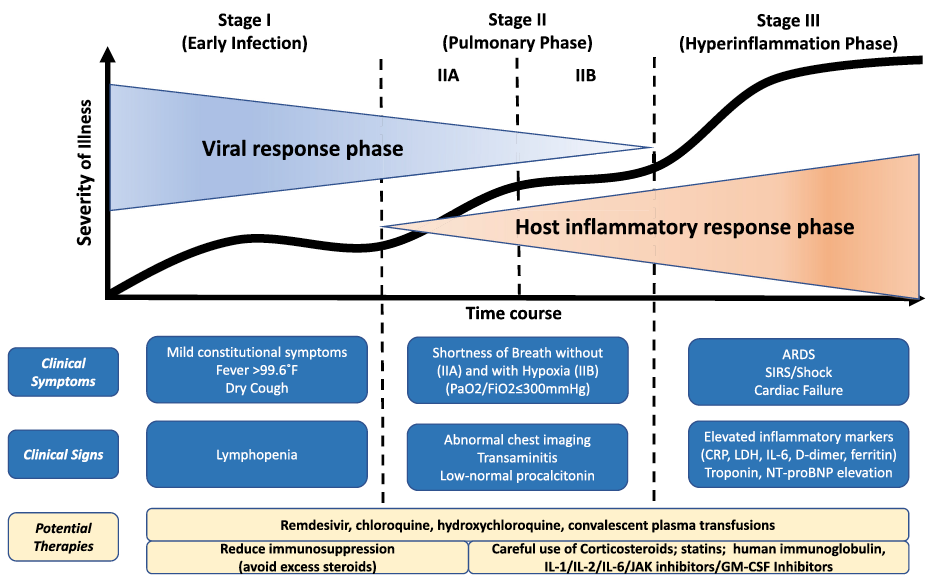
**

Reprinted from The Journal of Heart and Lung Transplantation, Vol 39/Issue 5, Siddiqi HK, Mehra MR, "COVID-19 illness in native and immunosuppressed states: A clinical-therapeutic staging proposal", pages 405-407, Copyright (2020), with permission from Elsevier.

**1.2 RATIONALE**

Currently, there is a compelling need for drugs with evidence-based efficacy and safety in patients with COVID-19. The need is particularly acute in patients with severe pneumonia in which cytokine release syndrome is triggered, as their prognosis is poor if there is a delay in starting treatment. In the context of health services being overwhelmed by excessive demand for intensive care and a limited availability of interleukin inhibitors, glucocorticoids are seen as a promising treatment option, and moreover, they are familiar to most practicing physicians. The aim of this study was to assess the safety and efficacy of glucocorticoids in COVID-19 pneumonia in the inflammation phase, specifically seeking to reduce ICU admissions, the need for mechanical ventilation and mortality.

**2.- HYPOTHESIS**

In patients with COVID-19 pneumonia in the inflammation phase (stage II), treatment with pulsed glucocorticoids is able to reduce the rate of treatment failure and other complications compared to the standard treatment.

**3.- OBJECTIVES**

**3.1 PRIMARY OBJECTIVE**

To assess the efficacy of pulsed glucocorticoids during the inflammation phase of the pneumonia caused by the SARS-CoV-2 virus.

**3.2 SECONDARY OBJECTIVES**

To compare

- 28-day mortality
- the proportion of patients admitted to the ICU at 28 days
- the proportion of patients requiring rescue therapy with tocilizumab at 14 days
- the length of hospital stay in days
- changes in laboratory parameters, namely, levels of C-reactive protein (CRP), interleukin 6 (IL-6), ferritin and D-dimer (DD)
- the proportion of patients experiencing serious adverse events
- the proportion of patients with bacterial, fungal and opportunistic infections
- viral clearance as assessed by nasopharyngeal PCR.

**4.- METHODS**

**4.1 STUDY DESIGN**

This is to be a phase 3 double-blind parallel-group randomised clinical trial:

1. Experimental group: standard of care + pulsed glucocorticoids versus
2. Control group: standard of care

The standard of care will consist of the treatment provided following the standard protocol at the time in the hospital. This may be updated during the course of the study.

**4.2 DISEASE/CONDITION**

Pneumonia due to SARS-CoV-2 virus (COVID-19).

**4.3 CONTEXT AND DURATION OF THE STUDY**

For this study, we will invite consecutive patients with a diagnosis of pneumonia caused by SARS-CoV-2 (COVID-19) to participate, recruiting them in the emergency department or on a hospital ward.

The study was originally conceived in the Hospital Complex of Navarra, but it was envisaged that other centres would be able to join the study, at the discretion of the study coordinator, and it has been extended to Hospital Moisès Broggi (Sant Joan Despí, Barcelona), researchers from this second hospital having been involved in the development of this version of the study protocol.

Each patient will be followed up throughout their stay on a hospital ward and to at least 28 days after the date of randomisation, any patients discharged from hospital earlier being contacted by phone. Patients will be censored when they reach the primary endpoint, are lost to follow-up or die. Patient recruitment will finish when we reach a fixed sample size. It is expected that patients will start to be included and followed up as of April 2020 and that follow-up will be completed in May 2020 [In fact, recruitment began on 28 May 2020 and is expected to be completed by February 2021]. Interim analysis of the results is due to be performed when recruitment reaches 50% of the fixed sample size.

**4.4 STUDY SUBJECTS**

The sample is to be selected from all patients who require hospital admission with a diagnosis of pneumonia due to SARS-CoV-2 based on reverse-transcription polymerase chain reaction (RT-PCR) of nasopharyngeal swabs or sputum in accordance with the recommendations of the Spanish Ministry of Health.

To be eligible, all patients must meet all the inclusion criteria and none of the exclusion criteria.

**Inclusion criteria:**

- Age ≥18 years
- Diagnosis of SARS-CoV-2 pneumonia confirmed by RT-PCR of nasopharyngeal swabs or sputum in accordance with the recommendations of the Spanish Ministry of Health
- History of symptoms compatible with COVID-19 ≥ 7 days
- At least one of the following: level of CRP >60 mg/L, IL-6 > 40 pg/ml and/or ferritin >1000 μg/L
- Provision of written informed consent.

**Exclusion criteria:**

- Allergy or contraindication to the drugs under study
- Signs of respiratory failure: SpO_2_ in ambient air < 90% or PaO_2_ in ambient air < 60 mmHg or PaO_2_/FiO_2_ < 300 mmHg
- Need for glucocorticoids or other immunosuppressants (including biologics) for another indication
- Decompensated diabetes mellitus
- Uncontrolled hypertension
- Psychotic or manic disorder
- Active cancer
- Pregnancy or breastfeeding
- Clinical or biochemical (procalcitonin > 0.5 ng/mL) suspicion of active infection with any pathogen other than SARS-CoV-2
- Management as an outpatient
- Conservative or palliative management
- Participation another clinical trial
- Any other major uncontrolled medical, psychological, psychiatric, geographic, or social problem contraindicating patient’s participation in the study or that hinders proper patient follow-up and adherence to the protocol and the evaluation of study outcomes.

**Criteria for study withdrawal:**

In accordance with the current version of the Declaration of Helsinki and legislation, patients have the right to withdraw from the study at any point and for any reason, without it affecting the medical care received by their doctor and/or referral hospital in the future. Any patients withdrawn will not be eligible for future inclusion in the study again.

Patients may be withdrawn from the study for any of the following **criteria for withdrawal:**

- Withdrawal of consent by the patient and/or refusal of treatment and/or lack of cooperation
- Toxicity, adverse events, or intercurrent illness that, in the opinion of the researcher, warrant withdrawal
- Development of any exclusion criteria which could be clinically relevant and affect patient´s safety or non-compliance with the protocol
- Decision of the principal investigator, if withdrawal from the study would be in the patient´s interests.

**5.- OUTCOME MEASURES**

5.1 PRIMARY ENDPOINT

Proportion of patients with treatment failure up to 14 days after randomisation.

Definition of treatment failure:

1. Patient death or
2. ICU admission or
3. Start of mechanical ventilation or
4. Clinical worsening, defined as:
   - Decrease in PaO_2_ by ≥15% from baseline + laboratory or radiological progression or
   - SpO_2_ in ambient air falling to < 90% or PaO_2_ in ambient air to < 60 mmHg or PaO_2_/FiO_2_ < 300 mmHg.

5.1 SECONDARY ENDPOINTS

- Mortality within 28 days after randomisation
- Proportion of patients admitted to the ICU within 28 days after randomisation
- Proportion of patients given tocilizumab as a rescue therapy within 14 days after randomisation
- Length of hospital stay in days
- Proportion of patients experiencing serious adverse events
- Proportion of patients with bacterial, fungal or opportunistic infections
- Changes in the laboratory parameters indicative of inflammation (CRP, IL-6, ferritin, and D-dimer) within 14 days after randomisation
- Viral clearance as assessed by nasopharyngeal RT-PCR at day 7.

Data collected will be entered into an electronic case report form; only study researchers will have access to these data and all the requirements in terms of data protection will be met.

**Dependent variables:**

- Death
- Date of death
- Rescue with an interleukin inhibitor, a biological therapy
- Date of rescue with interleukin inhibitor
- Need for mechanical ventilation (invasive or non-invasive)
- Date of mechanical ventilation (invasive or non-invasive)
- Need for high-flow oxygen therapy
- Date of high-flow oxygen therapy
- Need for ICU admission
- Date of ICU admission
- Date of ICU discharge
- Date of hospital discharge.

**Independent variables:**

Epidemiological data:

- Date of birth
- Sex.

Comorbidities:

- Exsmoker
- Smoker
- Type 2 diabetes mellitus
- Hypertension
- Hypercholesterolemia
- Kidney failure: stage 1-5
- Obesity: class 1-4
- Ischaemic heart disease
- Cerebrovascular disease
- Peripheral artery disease
- Chronic obstructive pulmonary disease
- Asthma
- Comorbidities as assessed by the Charlson index.

Clinical and hospitalisation-related parameters:

- Date of onset of symptoms
- Date of hospital admission.

Medication taken at home (prior to hospital admission):

- Metformin
- Dipeptidyl peptidase-4 inhibitors
- Sodium-glucose cotransporter-2 inhibitors
- Insulin
- Angiotensin-converting enzyme inhibitors- angiotensin receptor blockers
- Spironolactone
- Inhaled corticosteroids
- Hydroxychloroquine
- Others.

Medication given in hospital:

- Azithromycin, dates of starting and stopping treatment
- Lopinavir/ritonavir, dates of starting and stopping treatment
- Interferon beta-1b, dates of starting and stopping treatment
- Tocilizumab, dates of starting and stopping treatment
- Anakinra, dates of starting and stopping treatment
- Ciclosporin, dates of starting and stopping treatment
- Remdesivir, dates of starting and stopping treatment
- Colchicine, dates of starting and stopping treatment
- Other drugs, dates of starting and stopping treatment.

Baseline blood tests:

- SpO_2_/FiO_2_
- White blood cell count
- Lactate dehydrogenase (LDH)
- D-dimer
- Fibrinogen
- IL-6
- CRP
- Procalcitonin
- Ferritin
- Haemoglobin
- Platelet count.

Follow-up blood tests:

- SpO_2_/FiO_2_
- White blood cell count
- LDH
- D-dimer
- Fibrinogen
- IL-6
- CRP
- Procalcitonin
- Ferritin
- Haemoglobin
- Platelet count.

Radiological findings:

- Lobar pneumonia
- Multilobar pneumonia
- Radiological Severity Score for COVID-19.

Safety-related events (related to glucocorticoids):

- Decompensation of diabetes
- Infection
- Psychosis or mania
- Worsening of hypertension
- Dyspepsia
- Gastrointestinal haemorrhage
- Delay in viral clearance assessed by nasopharyngeal RT-PCR (7 days after the first RT-PCR)
- Others.

**5.- STUDY PROCEDURES**

**5.1 SCHEMATIC DIAGRAM OF THE STUDY**

**Figure 2. Flow of patients through the study**


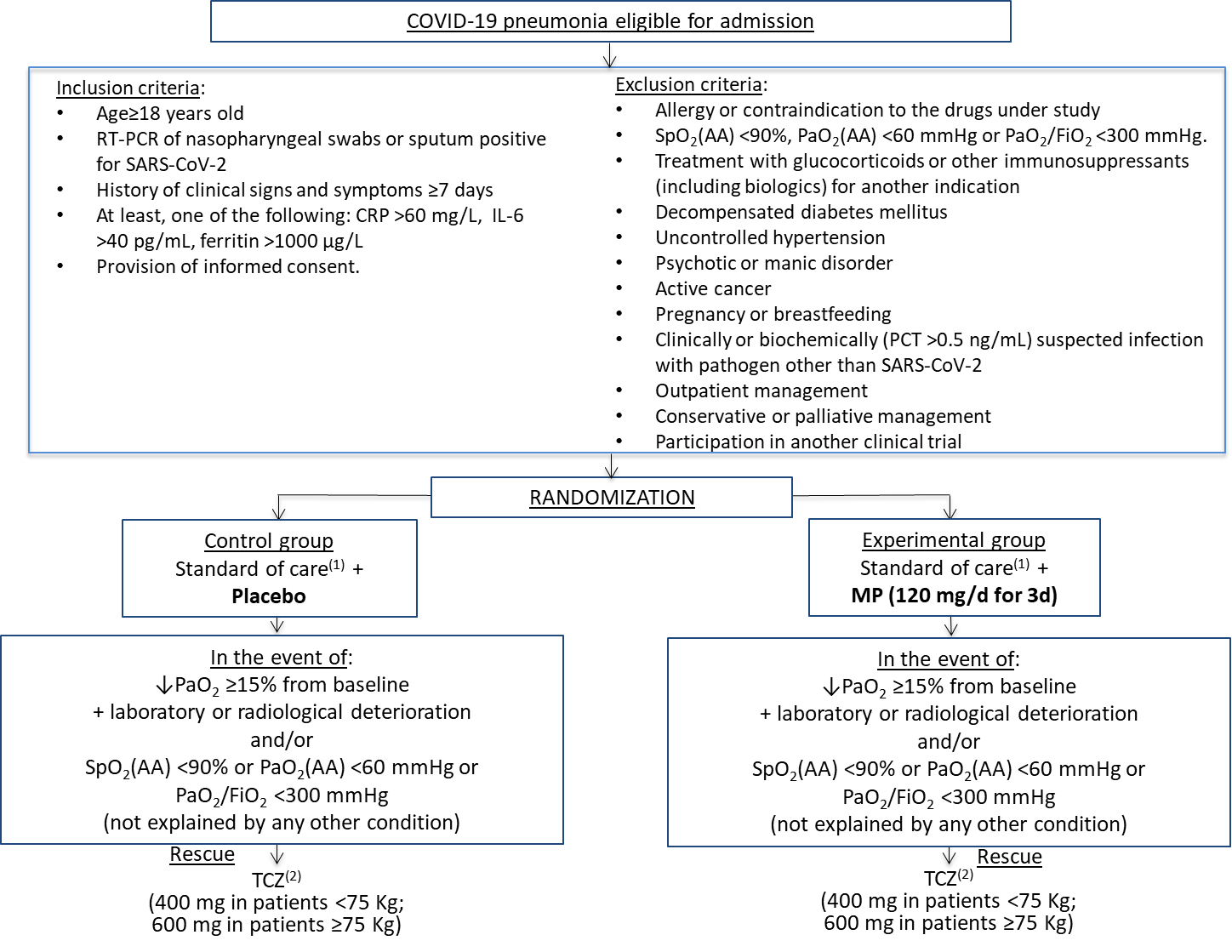


Abbreviations: RT-PCR, reverse-transcription polymerase chain reaction; CRP, C-reactive protein; IL-6, interleukin 6; SpO_2_(AA), oxygen saturation in ambient air; PaO_2_(AA), partial pressure of arterial oxygen in ambient air; PaO_2_/FiO_2_, ratio of arterial oxygen partial pressure to inspired oxygen fraction; PCT, procalcitonin; MP, methylprednisolone; TCZ, tocilizumab.

**^(1)^** standard of care is administered at the discretion of the researcher and may include: lopinavir/ritonavir: 200/50 mg orally, 2 tablets q12h for 7 to 14 days; ± remdesivir: 200 mg loading dose intravenously on day 1, followed by 100 mg/day for 5 to 10 days; or no drug treatment.

**^(2)^** Single dose of TCZ, dose may be repeated 12-24 hours after the first dose in the event of worsening in laboratory parameters, in accordance with the recommendations of the Spanish Agency of Medicines and Medical Devices.

Patients in both arms will be given omeprazole concomitantly.

Note: This base regimen may be modified in both arms if there are changes in standard of care at the participating hospitals.

**5.2 RANDOMISATION**

After obtaining patient informed consent, the treatment strategy will be decided by block randomisation stratified by age into two groups (<75 and ≥75 years old). Randomisation will be performed with the randomizeR package in R software, using two blocks sizes (sized 4 and 6), with random permutation. The sequence will be generated by a unit (the Clinical Trials Platform of Navarrabiomed) independent of the researchers who will carry out patient recruitment and implement the protocol. These researchers will be blinded to the arm to which patients are to be allocated until the start of the intervention. The Clinical Trials Platform will inform the hospital pharmacy concerning the group allocation of each patient included in the trial.

**5.3 INTERVENTION**

During the randomisation, patients with be randomly allocated to the experimental or control group (Figure 2):

- 1. Experimental group: standard of care at the discretion of the researcher, including lopinavir/ritonavir (200/50 mg orally, 2 tablets q12h for 7 to 14 days) ± remdesivir (200 mg loading dose intravenously on day 1, followed by 100 mg/day for 5 to 10 days), or no drug treatment, plus pulsed methylprednisolone (120 mg/day intravenously on days 1, 2 and 3).
  2. Control group (standard): standard of care at the discretion of the researcher, including lopinavir/ritonavir (200/50 mg orally, 2 tablets q12h for 7 to 14 days) ± remdesivir (200 mg IV on the first day, followed by 100 mg/day for 5 to 10 days), or no drug treatment, plus placebo* (intravenously on days 1, 2 and 3).

*Note: placebo consists of 100 ml of 0.9% sodium chloride.

The doses of methylprednisolone (120 mg IV) or placebo (NaCl 0.9% IV) will be prepared and dispensed by the hospital pharmacy. Only the staff responsible for preparing the medication will know patient group allocation. That is, neither the researcher nor nurses administering the treatment will know the content of the bags supplied.

Patients in both groups will be treated with tocilizumab if at any point the following are observed:

- Decrease in PaO_2_ ≥15% from baseline + laboratory or radiological deterioration and/or
- SpO_2_ in ambient air falling to < 90% or PaO_2_ in ambient air < 60 mmHg or PaO_2_/FiO_2_ below 300 mmHg not explained by any other condition.

**5.4 FOLLOW-UP METHODS**

**5.4.1 Baseline assessment**

All the patients included in the study will be assessed at baseline in the emergency department or on the hospital ward. This baseline assessment will include the following elements:

- Guided clinical history
- Medical history and comorbidities
- Concomitant treatments
- Vital signs: temperature, blood pressure, heart rate, respiratory rate and oxygen saturation
- Physical examination
- Blood tests: blood count, coagulation tests including D-dimer, renal function, and levels of CRP, procalcitonin, ferritin, LDH and IL-6
- Chest X-ray (interpreted by a pulmonary radiologist).

**5.4.2 Follow-up**

During hospital admission, patients will be followed-up in accordance with standard procedures for COVID-19 (11), with close monitoring of their vital signs (temperature, blood pressure, heart rate, respiratory rate, and oxygen saturation). All patients in the study will be assessed in person at least once a day by a doctor who is a member of a specialized COVID-19 team, in accordance with the established treatment protocol. The detection of any significant clinical change will prompt an addition assessment of vital signs and medical examination.

Blood tests involving a blood count, coagulation tests including D-dimer, and the measurement of renal function and levels of CPR, procalcitonin, ferritin, LDH and IL-6, will be performed every 48 hours and whenever there is a significant clinical change, at the discretion of the doctor in charge. Further, a chest X-ray will be performed every 48 hours and in the event of a significant clinical change, at the discretion of the doctor in charge, and the results will be interpreted by a pulmonary radiologist.

A follow-up RT-PCR will be performed before administrative discharge and this is to be performed at least 7 days after the first PCR.

**6.- STATISTICS**

**6.1 SAMPLE SIZE**

It was estimated that 60 patients (30 in each arm) were needed to detect as significant (two-tailed type I error of 0.05 and a type II error of 0.2) an absolute difference of 25% in the primary endpoint between the groups: 35% in the control group and 10% in the intervention group. Assuming a loss to follow-up of 20%, it is necessary to recruit a total of 72 patients.

**6.2 STATISTICAL ANALYSIS**

Initially, univariate descriptive analysis will be performed: calculating measures of central tendency and dispersion (means, standard deviation and confidence intervals or median and interquartile range depending on the distribution of the data) for continuous variables and frequencies for qualitative variables with their corresponding confidence intervals for the overall rates. Kolgomorov-Smirnov tests will be used to assess whether the continuous data follow a normal distribution and therefore parametric tests can be used.

Subsequently, it will be assessed whether randomisation achieved balance between the two arms: comparing the means of quantitative variables using Student’s t or the Mann-Whitney U tests, as appropriate, and qualitative variables using the chi-squared test or Fisher´s exact tests. If the desired balance is achieved, the primary endpoint will be compared between the groups to test the null hypothesis (H0) that the proportions are equal, assuming that the statistic for assessing the differences follows a normal distribution with a mean of 0 and standard deviation of 1; otherwise, multivariate analysis will be performed using logistic regression to assess the risk of treatment failure, estimating the effect of the intervention as an odds ratio with the corresponding 95% confidence interval.

The analysis will be performed on an intention-to-treat basis

**7.- SAFETY CONSIDERATIONS**

The E6(R1) Guidelines for Good Clinical Practice of the International Council for Harmonization of Technical Requirements for Pharmaceuticals for Human Use defines an adverse event as any unfavourable and unintended sign (including an abnormal laboratory finding), symptom or disease, whether or not related to the investigational product. This includes events related to the product, or the comparator, or to the procedures involved.

**7.1 SERIOUS ADVERSE EVENTS**

A serious adverse event is any untoward medical occurrence in a patient on trial medication that at any dose that

- results in patient death,
- is a life-threatening
- requires hospitalisation or prolongation of the current hospital stay
- results in persistent or significant disability/incapacity
- causes a congenital anomaly or birth defect
- is medically relevant.

Adverse events will be closely monitored until the condition resolves or is considered permanent, and at each appointment (or more often if required), the following will be assessed: any change in the severity, the suspected relationship with the drug of interest, and the interventions required to treat it and the outcome. All the adverse events (serious and non-serious) will be recorded in the corresponding electronic case report form of the study.

**7.2 PROCEDURE FOR REPORTING SERIOUS ADVERSE EVENTS**

The researcher will report any serious adverse events within the first 24 hours of learning of their occurrence to the person in charge of pharmacovigilance: Email: ruth.garcia.rey@navarra.es.

For this purpose, we will use the serious event report form. The person in charge of the pharmacovigilance will check the form received and request further information from the researcher as appropriate.

**8.- COMPLETION AND DISCONTINUATION OF THE CLINICAL TRIAL**

The clinical trial will end when all patients have completed the follow-up. Nonetheless, under certain circumstances, it may be halted at an earlier stage. Circumstances that my warrant termination of the trial include, but are not limited to:

- Identification of unexpected, significant or unacceptable risks to participants
- Inability to recruit an acceptable number of patients
- Non-compliance with the requirements of the protocol
- Non-compliance with the current good clinical practices or legislation.

**9.- DATA COLLECTION**

In this study, data are to be collected prospectively. The sources of information include patient’s electronic health records and data provided by doctors. All the information and documents associated with the study should be kept in the participating hospital.

Patients included in the study will be identified by a numerical code. In this way, the data collected by researchers will not include personal information, safeguarding patient confidentiality. The transmission, processing and dissemination of personal data from all the participating patients will be handled in accordance with Regulation (EU) 2016/679 of the European Parliament and of the Council of 27 April 2016 on the protection of natural persons with regard to the processing of personal data and on the free movement of such data, i.e., the General Data Protection Regulation.

**10.- ETHICAL CONSIDERATIONS**

**10.1 GENERAL CONSIDERATIONS**

This clinical trial will be carried out in accordance to the ethical principles of the most recent revision of the Declaration of Helsinki and current legislation. The original and revised versions of the protocol, information sheet and informed consent form have been approved by the clinical research ethics committee (CREC) of University Hospital La Princesa and the Spanish Agency of Medicines and Medical Devices (AEMPS) (approval of the latest version being granted on 28 August 2020, reference number: 17092020_08400, locator: QFSS36Q6C5).

**10.2 PATIENT INFORMATION AND INFORMED CONSENT FORM**

The researchers will be in charge of providing each patient or their legal representative with the patient information sheet and informed consent form. Individuals participating in the trial or their legal representative will be asked to sign and date the informed consent form prior to inclusion in the study, that is, before the performance of any intervention. The researcher will explain the nature of the trial, its purpose, the procedures involved, expected duration, and potential risks and benefits, as well as the fact that they have the right to withdraw at any time without giving a reason and without it affecting the medical care received. The researchers will give participants a copy of the patient information sheet and informed consent form and allow patients sufficient time to consider their decision about whether to participate in the trial.

In the event of amendments to the protocol that might directly affect the study participants, the information sheet and informed consent form will be modified to include the new information. Participants will then be asked to sign the new version of the form.

**10.3 CONFIDENTIALITY AND PERSONAL DATA PROTECTION**

The collection, processing and dissemination of personal data of patients participating in this study will be carried out in accordance with the current legislation on personal data protection, in particular in compliance with the General Data Protection Regulation, namely, Regulation (EU) 2016/679 of the European Parliament and of the Council of 27 April 2016. When collecting and processing patient personal data, all reasonable and appropriate measures will be taken to safeguard these data and preserve patient anonymity in relation to their health and medical records.

Participant identity will be coded in study documents, such that patient health records cannot be linked to an identified or identifiable person, and only authorised individuals (members of the research team, CREC or health authorities or the study monitor) for the proper performance of their duties, will have access to identifiable personal data.

At all times, the patient will be able to exercise the right, through the researcher, to access, rectify, erase or object to the use of their personal data.

**11.- DISSEMINATION OF RESULTS**

1. The results of the study will be published in scientific journals, mentioning the CREC that approved the study.

2. The anonymity of participating patients will be safeguarded at all times.

3. We will seek to publish the results and conclusions of this study in scientific journals before their dissemination to the general public.

**12.- REFERENCES**

1. World Health Organization. Director-General's remarks at the media briefing on 2019-nCoV on 11 February 2020. https://www.who.int/dg/speeches/detail/who-director-general-s-remarks-at-the-media-briefing-on-2019-ncov-on-11-february-2020 (Accessed on February 12, 2020).
2. Mehta P, McAuley DF, Brown M, et al. COVID-19: consider cytokine storm syndromes and immunosuppression. Lancet 2020;395(10229):1033–1034. doi:10.1016/S0140-6736(20)30628-0.
3. Chen C, Zhang XR, Ju ZY, He WF. Advances in the research of cytokine storm mechanism induced by Corona Virus Disease 2019 and the corresponding immunotherapies. Zhonghua Shao Shang Za Zhi 2020;36(0):E005. doi: 10.3760/cma.j.cn501120-20200224-00088.
4. Wang D, Hu B, Hu C, et al. Clinical Characteristics of 138 Hospitalized Patients With 2019 Novel Coronavirus-Infected Pneumonia in Wuhan, China. JAMA 2020. doi: 10-1001/jama.2020.1585.
5. Conti P, Ronconi G, Caraffa, et al. Induction of pro-inflammatory cytokines (IL-1 and IL-6) and lung inflammation by Coronavirus-19 (COVI-19 or SARS-CoV-2): anti-inflammatory strategies. *J Biol Regul Homeost Agents* 2020;34(2). pii: 1. doi: 10.23812/CONTI-E.
6. Russell CD, Millar JE, Baillie JK. Clinical evidence does not support corticosteroid treatment for 2019-nCoV lung injury. Lancet 2020;395(10223):473-475. doi: 10.1016/S0140-6736(20)30317-2.
7. WHO clinical management of severe acute respiratory infection when novel coronavirus (nCoV) infection is suspected. World Health Organization, Geneva. Jan 28, 2020.
8. Wu C, Chen X, Cau Y, et al. Risk factors associated with acute respiratory distress syndrome and death in patients with Coronavirus Disease 2019 pneumonia in Wuhan, China. *JAMA Intern Med* 2020. doi:10.1001/jamainternmed.2020.0994.
9. Zheng C, Wang J, Guo H, et al. Risk-adapted treatment strategy for COVID-19 patients. *Int J Infect Dis* 2020. doi: 10.1016/j.ijid.2020.03.047.
10. Buttgereit F, da Silva JA, Boers M, et al. Standardised nomenclature for glucocorticoid dosages and glucocorticoid treatment regimens: current questions and tentative answers in rheumatology. *Ann Rheum Dis* 2002;61(8):718-22.
11. Jin YH, Cai L, Cheng ZS, et al. A rapid advice guideline for the diagnosis and treatment of 2019 novel coronavirus (2019-nCoV) infected pneumonia (standard version). Mil Med Res 2020;7(1):4. doi: 10.1186/s40779-020-0233-6.

**APPENDIX IN RESPONSE TO THE MEASURES TAKEN BY THE SPANISH AGENCY OF MEDICINES AND MEDICAL DEVICES (AEMPS) REGARDING CLINICAL TRIALS OF HYDROXYCHLOROQUINE FOR COVID-19**

After the notification received by the researchers of the CORTIVID trial (EudraCT number: 2020-001827-15, Name of sponsor: Navarrabiomed-Fundación Miguel Servet) on 15 July 2020 and in accordance with the measures adopted by AEMPS on 22 June 2020 regarding clinical trials of hydroxychloroquine in COVID-19, the use of this drug in the CORTIVID trial was halted, as was the recruitment of patients on the drug. The design of the trial was modified accordingly, by amendment of the protocol to that detailed in this document.

The trial is currently in the active recruitment stage, with five patients having been included across two hospitals. [At the time of submission of this protocol for publication, 46 patients have been recruited and analysis is ongoing.]

We consider that the CORTIVID trial continues to be warranted and remains relevant in the current circumstances for the following reasons: 1) use of an active ingredient (methylprednisolone) and a different regimen (short three-pulse regimen) from that used in the RECOVERY trial; 2) research into the efficacy and safety of glucocorticoids in a clinical scenario that also differs from that considered in the RECOVERY study (patients with SARS-CoV-2 pneumonia not requiring oxygen but with poor prognostic factors); and 3) the possibility of confirming some of the findings of the RECOVERY trial in a double-blind randomized placebo-controlled study.
